# Preparation and Evaluation of Human-Murine Chimeric Antibody against Protective Antigen of *Bacillus anthracis*

**DOI:** 10.3390/ijms151018496

**Published:** 2014-10-14

**Authors:** Lina Hao, Feng Zheng, Siping Xiong, Dan Hu, Heng Lv, Qi Tang, Jin Yang, Zhenqing Feng, Changjun Wang, Jin Zhu

**Affiliations:** 1 Huadong Medical Institute of Biotechniques, Nanjing 210002, China; E-Mails: estherhaonj@163.com (L.H.); zhengf82@gmail.com (F.Z.); hudan1234@163.com (D.H.); lvheng570@gmail.com (H.L.); 2 Key Laboratory of Antibody Technique of Ministry of Health, Nanjing Medical University, Nanjing 210029, China; E-Mails: xiongsp_2011@163.com (S.X.); pippot@139.com (Q.T.); yangjin27@139.com (J.Y.); fengzhenqing@njmu.edu.cn (Z.F.)

**Keywords:** chimeric Fab antibody, anthrax, PA

## Abstract

The aim of this research is to develop a human/murine chimeric Fab antibody which neutralizes the anthrax toxin, protective antigen (PA). The chimeric Fab was constructed using variable regions of murine anti-PA monoclonal antibody in combination with constant regions of human IgG. The chimeric PA6-Fab was expressed in *E. coli*. BL21 and evaluated by ELISA and co-immunoprecipitation- mass spectra. The potency of PA6-Fab to neutralize LeTx was examined in J774A.1 cell viability *in vitro* and in Fisher 344 rats *in vivo*. The PA6-Fab did not have domain similarity corresponding to the current anti PA mAbs, but specifically bound to anthrax PA at an affinity of 1.76 nM, and was able to neutralize LeTx *in vitro* and protected 56.9% cells at 20 μg/mL against anthrax LeTx. One hundred μg PA6-Fab could neutralize 300 μg LeTx *in vivo*. The PA6-Fab has potential as a therapeutic mAb for treatment of anthrax.

## 1. Introduction

*Bacillus anthracis* is a Gram-positive spore-forming bacterium that causes the clinical spectrum of anthrax. Aerosolised distribution of spores can rapidly spread among a population of people and cause lethal inhalational anthrax due to the toxins produced during the replication of infectious bacilli [1]. Therefore, the employment of anthrax as a potential bioterrorism threat is very real and in 2001, an attack resulted in 22 confirmed cases, of which five were fatal [2].

Anthrax toxins comprise protective antigen (PA), lethal factor (LF) and edema factor (EF) [3]. PA63 is an active form of PA that is enzymatically processed from PA83, and oligomerizes to a haptamer. LF or EF confers toxicity only after binding to the haptamer to form lethal toxin (LeTx) or edema toxin (EdTx); therefore, PA plays a central role in the virulence of the pathogen [4]. Passive immunization of mAb has been an ideal therapeutic antibody treatment of anthrax due to its advantages over antibiotics treatment and vaccination [5,6,7,8]. However, murine mAb elicits detrimental alloantibody immune responses in humans [9,10]. Therefore, there are currently six clinically useful anti-PA mAbs, although only Raxibacumab [11], a human mAb, has been approved by USDA as a therapeutic anthrax mAb, in 2012. However, Raxibacumab binds poorly to PA with an affinity at 2.78 nM. Affinities of current anti-PA mAbs binding to receptor ranges from 0.17–33.3 nM, but an effective affinity for a mAb to bind to PA should be below this range [12]. Therefore, one anti-PA mAb may not be efficient enough to combat anthrax toxin, and instead, it may be that several anti-PA mAbs with different epitopes additively or synergistically targeting different domains of PA toxin are necessary for neutralization of PA [13,14].

Therefore, in this study, we prepared a chimeric human/murine Fab mAb combining variable regions of murine anti-PA mAb with human IgG constant regions and we evaluated the neutralizing capacity of PA6-Fab to neutralize LeTx *in vitro* and *in vivo*. The anti-PA PA6-Fab mAb may be a candidate treatment regimen for neutralization of PA and therapeutic treatment against anthrax.

## 2. Results and Disscution

### 2.1. Construction of PA-6-Fab Expression Vector

**Figure 1:**
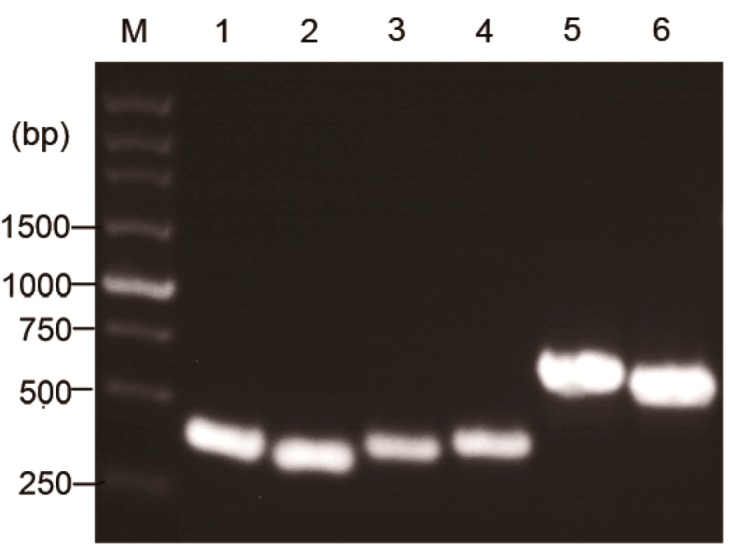
PCR products of PA-6-Fab expression vector. **M**: DNA marker; **lane 1**: murine variable regions of the heavy chain (V_H_); **lane 2**: murine variable regions of the light chain (V_L_); **lane 3**: human constant regions of the heavy chain domain 1 (C_H_1); **lane 4**: human constant regions of the light chain domain (C_L_); **lane 5**: heavy chain Fd; **lane 6**: light chain.

**Figure 2:**
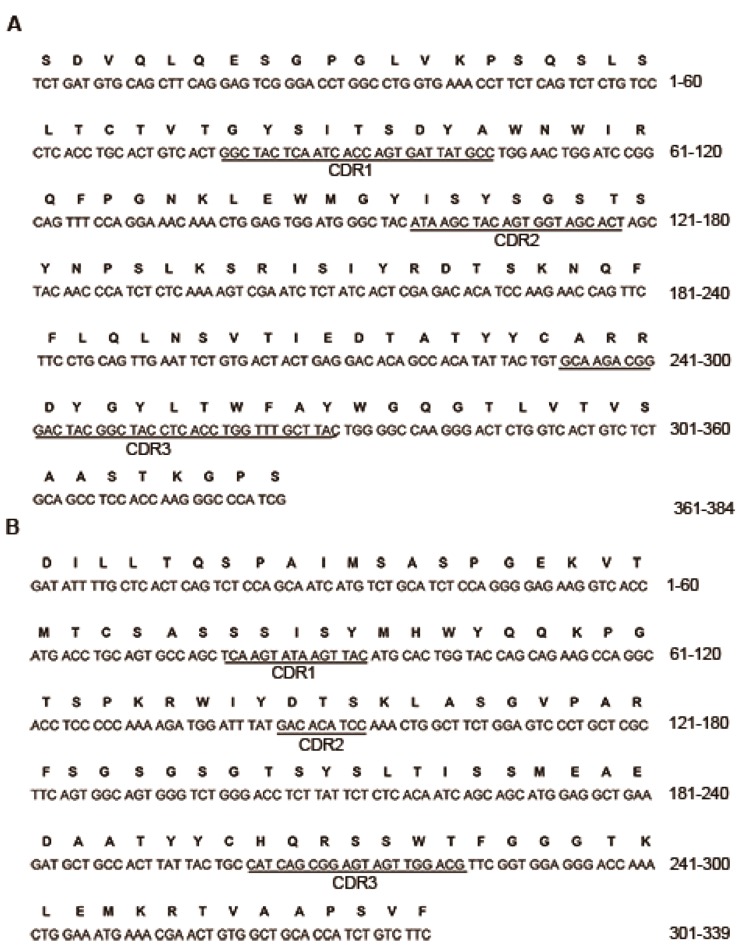
Nucleotide and deduced amino acid sequences of V_H_ and V_L_. The complementary determining regions (CDRs)are underlined, based on the analysis of VBASE2 database. (**A**) Nucleotide sequence of VH and deduced amino acid sequence of V_H_; (**B**) Nucleotide sequence of V_H_ and deduced amino acid sequence of V_L_.

Total RNAs were extracted from the PA6 hybridoma cells, and PA-6 cDNA was derived (Table 1). The murine V_H_ and V_L_ products were run on 1% agarose gel electrophoresis. As expected, V_H_ and V_L_ were about 380 and 360 bp, respectively. The correct V_H_ and V_L_ sequences were confirmed by sequencing analysis. Human C_H_1 and C_L_ products were about 390 and 420 bp, respectively. The chimeric heavy and light chains Fd were about 700 and 650 bp, respectively. All the PCR products are shown in Figure 1 and confirmed by sequencing analysis. The V_H_ sequence belonged to one member of the IGHV3 family, while the V_L_ sequence to IGKV4 subgroup. The complementary determining regions (CDRs) and framework regions (FRs) of V_H_ and V_L_ were determined by VBASE2 database (Figure 2). PCR indicated that the recombinant plasmid pET Duet-1/PA6-Fab was successfully constructed.

**Table 1:** Primer sequences.

Objection	Primer	Sequences
Murine variable regions of the heavy chain (V_H_)	VHF	5'-GCTGCCCAACCAGCCATGGCCGAGGTGCAGCTGGTGGAATCGGG-3'
	VHR	5'-CGATGGGCCCTTGGTGGAGGCTGCAGAGACAGTGACCAGAGT-3'
Murine variable regions of the light chain (V_L_)	VkF	5'-GGGCCCAGGCGGCCGAGCTCGATATTTTGCTCACTCAG-3'
	VkR	5'-GAAGACAGATGGTGCAGCCACAGTTCGTTTCATTTCCAGTTTGGTCCC-3'
Human constant regions of the heavy chain domain 1 (C_H_1)	CH1-F	5'-GCCTCCACCAAGGGCCCATCGGTC-3'
	dpseq	5'-GATATCAGAAGCGTAGTCCGGAACGTC-3'
Human constant regions of the light chain domain (C_L_)	HKC-F	5'-CGAACTGTGGCTGCACCATCTGTC-3'
	Lead B	5'-AAGCTTGGCCATGGCTGGTTGGGCAGC-3'
Chimeric heavy chain (Fd)	Lead H	5'-CATATGGCTGCCCAACCAGCCATGGCC-3'
	dpseq	Ditto
Chimeric light chain	RSF-F	5'-GGATCCGGAGGAGGAGGAGGAGGAGGCGGGGCCCAGGCGGCCGAGCTC-3'
	Lead B	Ditto

### 2.2. Expression and Purification of Human/Murine Chimeric PA6-Fab

**Figure 3:**
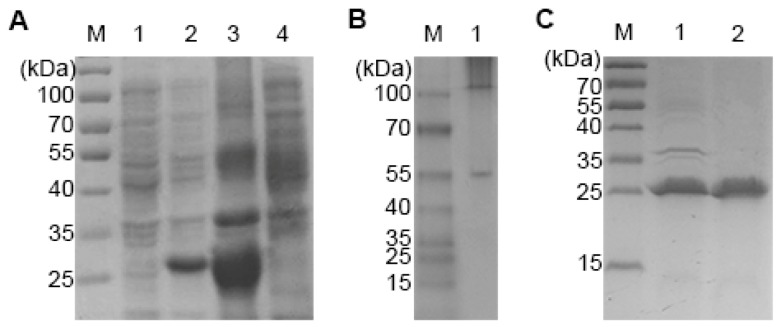
Expression and purification of PA6-Fab. (**A**) Expression of the PA6-Fab. **M**: protein marker; **lane 1**: supernatant of lysates; **lane 2**: pellet of lysates; **lane 3**: cell lysate of transfected BL21; **lane 4**: cell lysate of untransfected BL21; (**B**) Native polyacrylamide gel electrophoresis of the renatured PA6-Fab; (**C**) Affinity chromatography purified PA6-Fab. **M**: protein marker; **lane 1**: renatured PA6-Fab; **lane 2**: purified PA6-Fab.

The recombinant plasmid pET Duet-1/PA6-Fab was transformed into the *E. coli* strain BL21 (DE3). PA6-Fab expression was induced by addition of 1 mM isopropyl-β-d-thiogalactoside (IPTG) at 37 °C overnight. SDS-PAGE and Western blotting showed that both heavy chain Fd and light chain were expressed as the expected sizes and PA6-Fab was mainly found in the pellet of the lysate (Figure 3A). The inclusion body was denatured and gradually renatured. Native polyacrylamide gel electrophoresis demonstrated that heavy chain Fd and light chain were refolded correctly (Figure 3B). After purification by affinity chromatography, the purity of PA6-Fab reached 95% and the protein output approximated 2 mg purified protein from 1 L *E. coli* culture.

### 2.3. Binding Capability of PA6-Fab to PA

**Figure 4:**
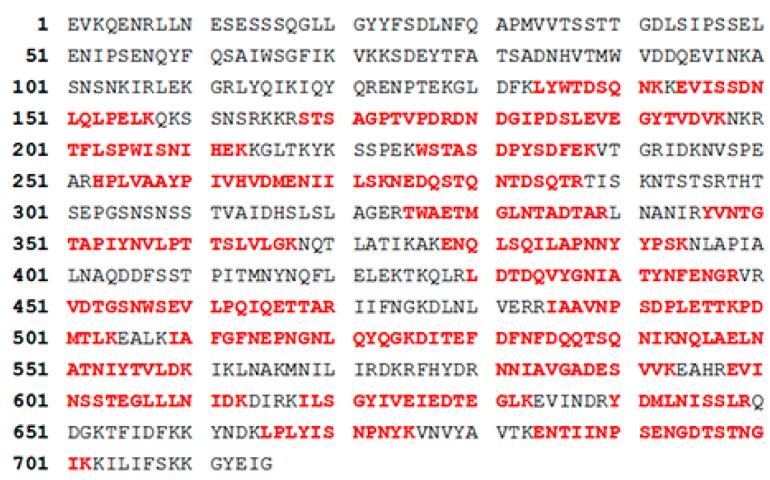
MS-based identification of anthrax protective antigen. The peptides were identified to match the PA sequence and are given in bold red.

Co-immunoprecipitation-mass spectra were used to evaluate the binding capability of PA6-Fab to PA. The relevant strips in the polyacrylamide gel electrophoresis were separated and detected by mass spectra. The detected protein sequence was also examined in Mascot software to derive 50% conformity with anthrax PA (Figure 4). These results further demonstrated that the chemeric PA6-Fab could identify anthrax PA specifically.

### 2.4. Analysis of Immunoreactivity of PA6-Fab to Anthrax PA by ELISA

**Figure 5:**
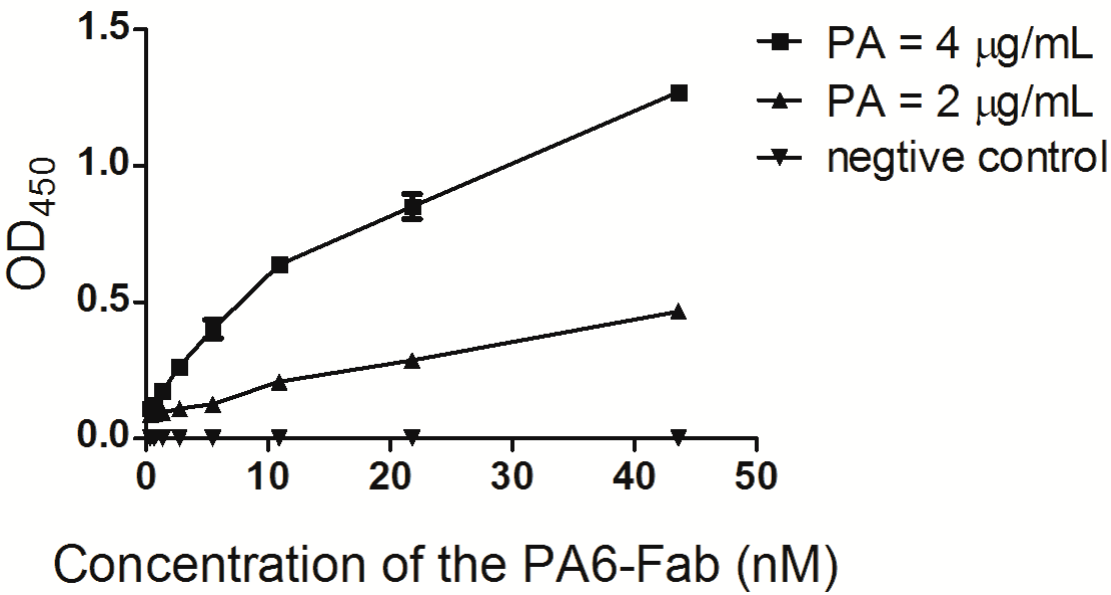
Immunoreactivity of PA6-Fab to anthrax PA. The immunoreactivity was measured by ELISA. The relationship of the concentration of the PA6-Fab and the absorbance at 450 nm were plotted by GraphPad Prism software 5.0 (GraphPad Software, San Diego, CA, USA).

The immunoreactivity of the PA6-Fab was assessed by ELISA. The ELISA signal correlated with the values at absorbance at 450 nm in as dose dependent manner (Figure 5). This result indicated that PA6-Fab could identify PA specifically. [Ab’]t was 1.21 nM and [Ab]t was 0.66 nM. According to the equation Kaff = 1/(2[Ab’]t − [Ab]t), the Kaff of the PA6-Fab was 5.07 × 10^−9^ L/mol (Kd = 1.76 nM).

### 2.5. In Vitro and in Vivo Neutralization Assay of PA6-Fab to LeTx

**Figure 6:**
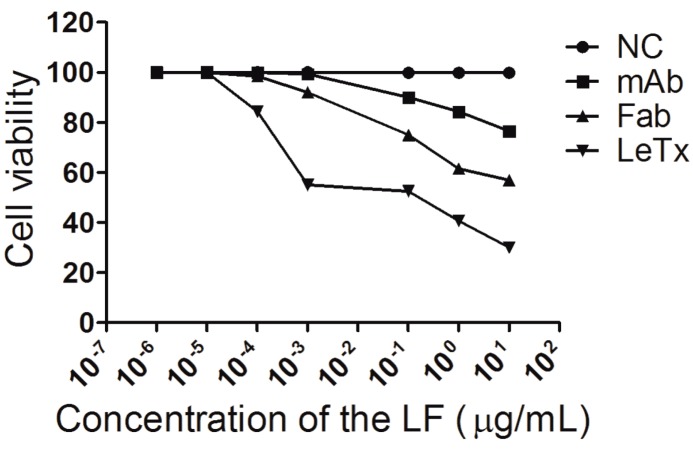
Anthrax LeTx neutralization activity assay of PA6-Fab. The cell protection using 20μg murine anti-PA or the PA6-Fab was detected at different concentrations of LeTx. Cells cultured in DMEM alone or with LeTx were used as controls.

Different concentrations of LeTx and 20 μg PA6-Fab were mixed and added to mouse macrophage J774A.1 cells. Neutraliztion capability *in vitro* demonstrated that PA6-Fab protected J7741A.1 cells against LeTx. At LF 10 μg/mL, approximately 56.9% of the cells were protected with PA6-Fab at 200 ng/mL, and 76.5% of the cells were protected by the murine monoclonal antibody (Figure 6). For *in vivo* assays, the rats of control group and 50 μg PA6-Fab group showed toxic symptom and died 3 h after intravenous injection. The rats of 100 μg or above dosage PA6-Fab group were alive and had no obvious symptoms.

### 2.6. Discussion

In this study, we combined variable regions of anti-PA mAb with constant regions of human IgG to prepare a chimeric Fab with supposed high affinity and low immunogenicity. CDRs and FRs of V_H_ and V_L_ were analyzed and the V_H_ sequence was a member of the IGHV3 family, while the V_L_ sequence belonged to the IGKV4 family. The V_L_ sequence was 99% similar to mus musculus immunoglobulin kappa chain complex (Igk) on chromosome 6. The V_H_ sequence was 97% similar to the mus musculus clone LBT5PaTM9 immunoglobulin mu heavy chain variable region mRNA. The PA6-Fab domain was not found to have similarity to the existing anti-PA mAbs after sequence comparison (NCBI). Therefore, the PA6-Fab mAb enriches the current anti-PA mAb profile and may be a candidate anti-PA mAb for combining several anti-PA mAbs with different epitopes to additively or synergistically target different domains of PA toxin [9,15,16]. We utilized co-immunoprecipitation-mass spectra to investigate whether human constant regions of human IgG influenced Fab antigenicity. The amino acid sequence of PA which bound to PA6-Fab was also examined in Mascot software, and 50% of the amino acid sequences of the PA6-Fab-PA complex conformed with anthrax PA, indicating that PA6-Fab could interact with PA.

The neutralization assay showed that PA6-Fab at only 200 ng/mL could totally protect cells at a low concentration of LeTx (PA, 100 ng/mL; LF, 0.1 ng/mL) and 56.9% of cells at 10 μg/mL of LF, while 76.5% of the cells could be protected with 200 ng/mL murine mAb PA6. *In vivo* 100 μg PA6-Fab could protect rats when 300 μg LeTx was injected. While 1–10 μg of other mAbs could neutralize 100% of LeTx activity on macrophages when PA83 was 500 ng/mL and LF was 1 μg/mL, 1.5 mg of mAbs could neutralize 200 μg rPA plus 200 μg rLF [17,18,19]. These results demonstrated that PA6-Fab could neutralize LeTx and protect cells and rats. The non-competitive ELISA using PA antigen demonstrated that the PA6-Fab could identify PA in a dose-dependent manner. The PA6-Fab monoclonal antibody had a binding affinity of 1.76 nM to LeTx which falls within the current monoclonal anti-PA binding affinity from 0.1–33.3 nM and higher than the binding affinity of Raxibacumab binding affinity to PA, which is 2.78 nM [5]. Because the human/murine chimeric Fab monoclonal antibody was expressed by prokaryotic expression system whose processing and modification systems are imperfect, the biological activity of Fab antibody may be affected [20]. Fab antibody is a small molecule (1/3 molecular weight of completely IgG) and has only half of antigen binding sites of complete IgG [21], so its affinity was still high although a little lower than IgG antibody. However, even though it is not a complete/full-length chimeric IgG, the affinity is higher for the chimeric Fab fragment than for the murine Fab fragment; the reason for this is that the variable region is amplified from the murine antibody PA6 which has the high affinity.

The human constant regions may change the conformation of the Fab regions of murine mAb affecting its ability to bind antigen [22]. However, the PA6-FAb in this study had a greater affinity to LeTx; therefore, the mechanism of how PA6-Fab binds and neutralizes LeTx remains to be further investigated in future studies.

## 3. Materials and Methods

### 3.1. Materials

Anthrax protective antigen (PA83) and lethal factor (LF) was provided by the Chinese Center for Disease Control and Prevention (CDC). The PA6 hybridoma cells and murine mAb against anthrax protective antigen (PA6) were developed and purified in our lab. The mouse macrophage cell line J774A.1 was purchased from American Type Culture Center, USA (Manassas, VA, USA). Plasmid pComb 3XTT was used to amplify constant regions of the heavy chain domain 1 (C_H_1) and the light chain (C_L_) of humanized IgG.

### 3.2. Construction of PA6-Fab Expression Vector

Total RNA was extracted from the PA6 hybridoma cells using a Promega RNA extraction kit according to the manufacturer’s protocols. PA6 cDNA was amplified with Prime Script™ RT Master Mix of Takara in Dalian, China. The primers were designed and synthesized in accordance with described previously [23]. Variable regions of the heavy chain (V_H_) and the light chain (V_L_) of the mAb were amplified by PCR from PA6 cDNA. The PCR products were gel-purified and extracted using a gel extraction kit from Qiagen (Valencia, CA, USA). The Purified V_H_ and V_L_ PCR products were ligated to a T vector and sequenced. The sequences were further analyzed using the VBASE2 database [24]. Then constant regions of the heavy chain domain 1 (C_H_1) and the light chain (C_L_) of humanized IgG were amplified. 100 ng of each V_H_ and C_H_1 PCR products were used in overlap PCR to create the heavy chain (Fd) and 100 ng of each VL and CL PCR products were used to create the light chain. Chimeric light and Fd chains were inserted into pET Duet-1 plasmid successively. All constructs were confirmed by sequencing.

### 3.3. Expression and Purification of Human/Murine Chimeric PA6-Fab

The recombinant plasmid pET Duet-1/PA6-Fab was transformed into competent *E. coli* BL21 (DE3) cells. 1 mM isopropyl-β-d-thiogalactoside (IPTG) was added to 200 mL cultures to induce production of PA6-Fab at 37 °C. The cells were collected and resuspended in lysis buffer 20 mL (10 mM Tris, 2 mM EDTA and 1 mL/L Triton-X 100, pH 8.0). After sonication with 300 W power for 30 min, the lysate was centrifuged at 10,000× *g* for 10 min, and the supernatant and pellet were subjected to SDS-PAGE (12%), stained with Coomassie blue, and examined by Western blotting using goat anti-human Fab IgG. The inclusion body was denatured with 8 M urea solution 10 mL and dialyzed with 100 mM Tris buffer (pH 8.5) down a urea gradient with 6, 4, 2, and 0 M urea. The renatured PA6-Fab was examined by 12% native polyacrylamide gel electrophoresis. Purification was performed by Protein L affinity chromatography. Protein was binded with binding buffer (Na_2_HPO_4_ 0.05 M, NaH_2_PO_4_ 0.05 M, NaCl 0.35 M, pH 7.2), then eluted by elution buffer (glycine 0.1 M, NaCl 0.15 M, pH 2.8). PA6-Fab was neutralized with 0.1 M Tirs and ultrafiltrated. The purity of PA6-Fab was examined by SDS-PAGE with Coomassie blue staining.

### 3.4. Co-Immunoprecipitation and Mass Spectra Analyses of PA6-Fab Binding to PA

To confirm the ability of PA6-Fab to neutralize anthrax PA, we conducted co-immunoprecipitation-mass spectra analysis. The bacteria were lyzed as mentioned previously. Lysate of bacterial expressed PA6-Fab (30 μL) was incubated with 100 μL murine anti-PA (4.0 mg/mL) or the PA6-Fab (2.31 mg/mL) at 4 °C for 5 h. Fifty μL protein L agarose was added to the mixture of lyzed bacteria and/or the antibody and incubated at 4 °C overnight. Mixture with L agarose was washed three times with PBST and resuspended with 50 μL PBS. The resuspension solution (10 μL) was analyzed by SDS-PAGE, and the gel was stained with Coomassie blue. The PA6-Fab resuspension and the murine anti-PA resuspension solutions were subjected to Western blotting using murine anti-PA and rabbit anti-PA, respectively. The relevant strips from the polyacrylamide gel electrophoresis were separated and detected by mass spectra.

### 3.5. PA6-Fab ELISA

The PA6-Fab immunoreactivity was assessed by ELISA. 96-well microtiter plates were coated with 100 μL PA (4 or 2 μg/mL) overnight at 4 °C and blocked with 1% (wt/vol) BSA in PBS plus 0.1% Tween-20 (PBST, pH 7.4) at 37 °C for 1 h and washed three times with PBST. Bound PA6-Fabs were incubated with peroxidase-conjugated goat anti-human IgG at 37 °C for 60 min, followed by color development using TMB peroxidase substrate, and absorbance was read at wavelength 450 nm with an ELISA reader. The concentrations of PA-6 Fab and the absorbances were plotted to two hyperbolic curves by GraphPad Prism software version 5.0 (GraphPad Software, San Diego, CA, USA). Affinity constant (K_aff_) was calculated using SPSS statistical software (version 13.0) by equation K_aff_ = 1/(2[Ab’]t − [Ab]t), where [Ab’]t is the free mAb concentration at the OD_50_ of 2 μg/mL coated antigen, and [Ab]t was the free mAb concentration at the OD_50_ of 4 μg/mL coated antigen.

### 3.6. In Vitro and in Vivo Neutralizing Capability of PA6-Fab

The capacity of the PA6-Fab to neutralize LeTx *in vitro* was measured by toxin neutralization activity. The mouse macrophage cell line J774A.1 (10^4^ cells/well) was seeded in 96-well microtiter plates overnight. LF (10 μg/mL) 100 μL were 10-fold serially diluted and mixed with PA (0.1 μg/mL) and 100 μL murine anti-PA or the PA6-Fab both at 200 μg/mL was added to the diluted LeTx. Cells cultured in DMEM alone were used as the baseline of percentage of cell death, while cells cultured in DMEM with LeTx were used as a negative control. Cells were continuously cultured at 37 °C for another 3 h to calculate the percentage of cell death. The potency of PA6-Fab to neutralize LeTx was assumed as proportional to the percentage of cell death. The concentration of LF and the percentage of cell viability were plotted by GraphPad Prism software.

For *in vivo* assays, Fisher 344 rats (female, 6 weeks old, 160 to 180 g) were selected through a laboratory animals screening. Lethal dose of LeTx to the body was measured, 50, 100, 150, 200, 250, 300, 350, 400 μg LeTx was injected into rats separately. The best lethal dose of LeTx (300 μg PA and 300 μg LF) was intravenously injected into Fisher 344 rats, followed by intravenous injection of 1000, 500, 250, 150, 100 or 50 μg PA6-Fab or PBS only, once a day for total of 7 days. Morbidity of rats was recorded.

## 4. Conclusions

In summary, we have successfully prepared a human/murine chimeric Fab monoclonal antibody, which can bind the anthrax PA toxin with greater specific affinity and can neutralize anthrax LeTx *in vitro* and *in vivo*. The PA6-Fab mAb antibody may potentially be used alone or in combination with other anti-PA mAbs for therapeutic treatment of anthrax.
